# Effects of a manualized short-term treatment of internet and computer game addiction (STICA): study protocol for a randomized controlled trial

**DOI:** 10.1186/1745-6215-13-43

**Published:** 2012-04-27

**Authors:** Susanne Jäger, Kai W Müller, Christian Ruckes, Tobias Wittig, Anil Batra, Michael Musalek, Karl Mann, Klaus Wölfling, Manfred E Beutel

**Affiliations:** 1 Outpatient Clinic for Behavioural Addictions, Department of Psychosomatic Medicine and Psychotherapy, University Medical Center of the Johannes Gutenberg University Mainz, Mainz, Germany; 2 Interdisciplinary Center for Clinical Trials (IZKS), University Medical Center of the Johannes Gutenberg University Mainz, Mainz, Germany; 3 Section Addiction Medicine and Addiction Research, University Hospital Tübingen, Tübingen, Germany; 4 Anton-Proksch Institute Wien, Wien, Austria; 5 Addiction Medicine, Central Institute of Mental Health Mannheim, Mannheim, Germany

**Keywords:** Internet addiction, computer game addiction, STICA, intervention, cognitive behavioral therapy

## Abstract

**Background:**

In the last few years, excessive internet use and computer gaming have increased dramatically. Salience, mood modification, tolerance, withdrawal symptoms, conflict, and relapse have been defined as diagnostic criteria for internet addiction (IA) and computer addiction (CA) in the scientific community. Despite a growing number of individuals seeking help, there are no specific treatments of established efficacy.

**Methods/design:**

This clinical trial aims to determine the effect of the disorder-specific manualized short-term treatment of IA/CA (STICA). The cognitive behavioural treatment combines individual and group interventions with a total duration of 4 months. Patients will be randomly assigned to STICA treatment or to a wait list control group. Reliable and valid measures of IA/CA and co-morbid mental symptoms (for example social anxiety, depression) will be assessed prior to the beginning, in the middle, at the end, and 6 months after completion of treatment.

**Discussion:**

A treatment of IA/CA will establish efficacy and is desperately needed. As this is the first trial to determine efficacy of a disorder specific treatment, a wait list control group will be implemented. Pros and cons of the design were discussed.

**Trial Registration:**

ClinicalTrials (NCT01434589)

## Background

The internet has become accessible for the great majority of the population (for example flat rates, WLAN, or portable computers). In a representative German sample (n = 2475) in 2009 the rate of leisure-time internet users for women was about 51% and for men about 60%. The most frequently used internet applications were email (93%), information and research (92%), shopping (76%), and chatting (62%) [1]. In 2004 about 68% of American adults used the internet regularly and 4% to 14% showed one or more markers of problematic use with a prevalence of internet addiction (IA) at about 1% [2], which is concordant with an actual German study [3]. The onset of manifest addicted behavior is reported in the late 20s or early 30s age groups [2]. In epidemiological studies, prevalence rates of addicted internet use and computer game behavior range between 1.5% to 3.0% in German [3,4] and Austrian [5] adolescents, respectively.

According to Block [6], three subtypes of IA/computer game addiction (CA) (excessive gaming, sexual preoccupations, and email/text messaging) have four components in common: (a) excessive use (along with a loss of sense of time or the ignorance of basic drives); (b) withdrawal (for example tension, anger, agitation, and/or depression when access to a computer is blocked; (c) tolerance (increasing use or sophistication of computer equipment); and (d) negative repercussions (for example poor achievement/performance, fatigue, social isolation, or conflicts).

Salience, mood modification, tolerance, withdrawal symptoms, conflict, and relapse are additional diagnostic criteria for IA and CA [7]. The addicted individual is increasingly attracted towards the excessive behavior and life is emotionally and cognitively preoccupied with the application (for example computer game), requiring more and more time in order to regulate his mood states. Empirical studies [4,8,9] have demonstrated that the symptom complex of IA/CA [10,11] matches the criteria of substance disorders. Results of neurobiological studies have identified neurophysiological mechanisms in IA/CA equivalent to substance abuse (alcohol [12] and cannabis addiction [13]).

Patients with CA and IA have increasingly sought help in addiction counseling [14], because of serious negative psychosocial consequences (social, work/education, health) which have been documented along with high mental co-morbidities [15-19]. IA is strongly associated with dimensionally measured depression [18,20], indicators of social isolation or behavioral deficits (for example ADHD [18,21,22]), or impulsivity [23]. In the Grüsser-Sinopoli outpatient clinic for behavioral addiction, from 2008 to 2010, a total of 326 patients have been assessed for IA/CA by clinical examination and tests. Of those, 192 patients were classified as IA/CA. They were predominantly (97%) male and aged from 18 to 30 years. They showed strong evidence of social phobia and depression as well as performance decrements in school and work.

Despite its increasing importance as a major health problem among adolescents and young adults at present, there is still a lack of evidence-based interventions for IA/CA. Preliminary evidence has only been generated in open trials for non-European and Asian populations [24,25].

Therefore, a specific short-term treatment program for IA/CA, based on cognitive behavior therapy (STICA) was developed. A preliminary evaluation of the manualized STICA treatment was performed in an open trial of the Grüsser-Sinopoli outpatient clinic for behavioral addiction with a total of 33 patients. Twenty-four out of this sample completed STICA regularly, nine patients terminated treatment prematurely and were considered as drop-outs (27%). Based on the full sample of 33 patients (intent to treat analysis) criteria for treatment response (primary efficacy endpoint) were reached by 67% which corresponds to a large effect size of 1.27 [Wölfling K, Müller KW, Beutel ME: Treatment outcome of a manualized cognitive behavior therapy in Internet and Computer game addiction, unpublished].

This study will assess the efficacy of the manualized STICA. Furthermore, the durability of treatment response in these patients and the impact on associated psychiatric symptoms (for example social anxiety and depression) will be determined. Currently STICA is the only manualized outpatient treatment program for IA/CA in Germany [26] and further international concepts and clinical trials were not methodologically convincing [27].

## Methods/Design

### Study centers

This multicenter study is coordinated by the outpatient clinic for behavioral addictions of the Clinic for Psychosomatic Medicine and Psychotherapy of the University Medical Center Mainz. Three centers will further participate, the Anton-Proksch-Institute, Austria, the Section Addiction Medicine and Addiction Research of the University Hospital Tübingen, and the Addiction Medicine of the Central Institute of Mental Health in Mannheim.

Investigators in all centers are psychotherapists (physicians or psychologists) and experts in the treatment of addiction behavior.

### Participants

Patients will be included, if the following eight inclusion criteria are fulfilled: (1) IA/CA according to the AICA (Assessment of Internet and Computer game Addiction) expert rating for at least 6 months and (2) a score ≥ 7 in the AICA self-report IA/CA. (3) Patients with co-morbid disorders will be included, provided that IA/CA is the primary diagnosis. The study will only include (4) men in (5) the age between 17 and 45 years. (6) If the patients are currently on psychotropic medications, no changes in medications and dosages in the past 2 months and during STICA treatment are allowed. (7) If currently off all psychotropic medications, patient must have been off at least 4 weeks. (8) During STICA no other ongoing psychotherapy is allowed and previous psychotherapy must have been completed for at least 4 weeks.

Patients with a score <40 in the Global Assessment of Functioning (GAF [28]) or severe major depression (Beck Depression Inventory; BDI-II [29] ≥ 29) are excluded. Additional exclusion criteria are current alcohol or drug addictions, borderline, antisocial, schizoid, and schizotypal personality disorders, a lifetime diagnosis of schizophrenia, schizoaffective, bipolar, or organic mental disorder and a current unstable medical illness.

Over a time period of 36 months we plan to include 192 patients in the study. The patients will be randomly assigned to the intervention or to the wait list control group (WLC). Prior to randomization, a total of 18 patients have to be allocated to the trial. The intervention group will start treatment immediately after randomization, whereas the WLC group has to wait over a period of 4 months, until they will receive the same therapy.

### Intervention

The manualized STICA [26] is based on a cognitive behavior approach and combines group with individual therapy. STICA comprises 23 psychotherapy sessions with a total duration of 4 months. Fifteen out of twenty-three sessions will be weekly group sessions (100 min each) and eight will be fortnightly individual sessions (50 min).

Table 1 shows treatment phases and strategies during the early, middle, and termination phases. Based on understanding the mechanisms and the consequences of IA/CA (early phase), patients are trained to identify the triggers of their own dysfunctional internet use. By using diaries, social skill training, and exposition training, patients learn to reduce and to control their computer and internet use. In the termination phase of treatment, tools will be transferred to daily life and strategies for relapse prevention will be discussed.

**Table 1:** STICA Treatment phases and strategies

**Treatment phase**	**Key interventions**
Early phase	Patient education on mechanisms and effects of IA/CA (for example learning theories, development and consequences of IA/CA, vicious cycles of addiction, and so on)
	Promotion of social communication and bonding in the group and individual setting
Middle phase	Identification of the triggers of dysfunctional internet use (for example emotional states, maladaptive cognitions, daily hassles, and so on) by keeping diaries
	Functional analysis of the addictive behavior
	Enabling functional internet use by appropriate problem-solving strategies
	Assisting the establishment of a social network in real life
	Building alternative activities
	Self-monitoring to reduce procrastination tendencies
	Promotion of social communication
	Exposition training (confronting and deleting access to critical applications, for example the self-created avatar)
	Skill training (for example coping with stress and problems, social skills, building alternative activities, and so on)
	Promotion of functional computer and internet use
Termination and relapse prevention	Review of transfer of treatment tools to daily life
	Functional computer/internet use
	Elaboration of tools preventing a relapse

### Assessment

**Figure 1:**
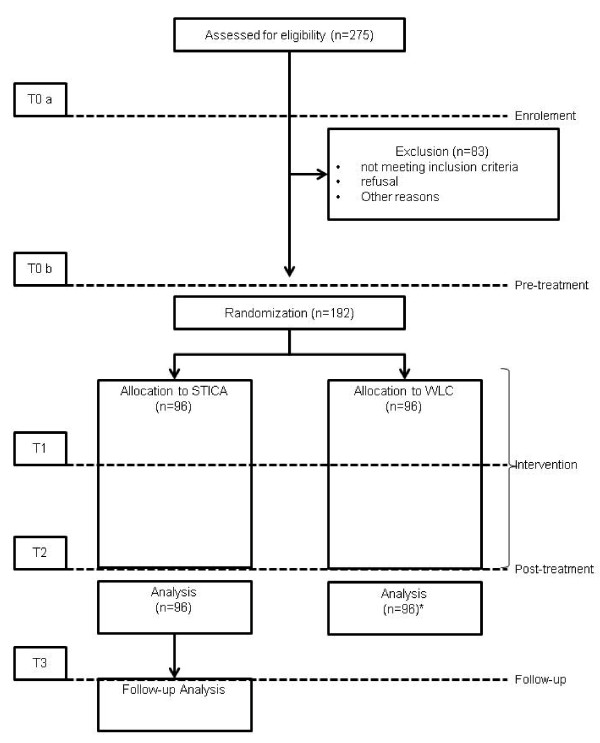
**Flow chart of the study.** Patients of the wait list control group (WLC) will be offered STICA treatment after the intervention group has finished. The follow-up analysis will be performed separately for WLC.

Figure 1 shows a flow chart of the five time points of assessment. At T0a patients are informed about the study and assessed for eligibility. Patients fill out the AICA-S [30,31], Müller KW, Glaesmer H, Brähler E, Wölfling K, Beutel M; Internet addiction in the general population. Results from a german population-based survey. unpublished] and the BDI-II [29]. AICA-S values range between 0 and 27, and scores ≥ 7 have been defined as problematic internet use. Therapists assess onset, course, and criteria of IA/CA, treatment history, motivation for therapy, and the GAF [28]. The AICA Checklist will be rated by an independent and blinded rater.

The assessment T0b is performed immediately before randomization and commencement of treatment. The criteria for IA/CA will be rechecked by self-report measurements, if the delay due to the group recruitment exceeds 2 weeks. Therapists fill out the GAF [28] and collect information about medications, other therapies, and treatment history. An independent and blinded rater will assess mental disorders with the SCID-I/II [32] and conduct the AICA-Checklist. A drug screening is further used to assess objective information on drug consumption. Self-report assessment includes IA/CA (AICA-S [30,31]), depression (BDI-II [29]), obsessive-compulsive behavior (SCL-90-R [33]), generalized anxiety and panic (Patient Health Questionnaire [34]), somatization [34], general distress [34], depersonalization (CDS-2 [35]), and social fear (LSAS [36]). Patients also fill out dimensions of personality (NEO-FFI [37]), attention deficit disorder (WURS-k [38]), self-efficacy (SWE [39,40]), positive and negative affectivity (PANAS [41]), and adverse childhood experiences (ACE [42]). Finally, they answer questions concerning perceived stress (PSS [43]) and about their life satisfaction (FLZ [44]). Attention of the patients will be checked with the d2 [45].

After 2 months of therapy (T1) patient-rated outcome measures are reapplied (AICA, GAF, BDI-II) and drug screening repeated. The outcome measures are supplemented by assessments of group climate (GCQ [46]) and therapeutic alliance (HAQ [47]).

Immediately after completion of the intervention (T2) patients fill out a set of questionnaires identical with the set at T0b, except for the trait measures (NEO-FFI, WURS-k, ACE). Group climate and therapeutic alliance are assessed additionally. Drug screening is applied and is obligatory. For patients of the WLC group this is the final assessment. Shortly after the survey their intervention will start.

Patients of the intervention group are asked to evaluate the stability of treatment effects 6 months after termination of treatment (T3). Thereby the used set of questions corresponds to T2.

### Data collection

In this study there are two sources of electronic study data. An eCRF has been developed for the investigators to document their study data in a database stored, maintained, and administered by the IZKS Mainz. It is password protected with individual accounts for all investigators.

Patients will answer the self-report questionnaires by entry forms customized for iPADs. Each patient receives only access to his own current questionnaire.

After data collection, eCRF and iPAD data will be transformed into one SAS database for evaluation.

### Objectives and hypotheses

The purposes or this study are to determine the efficacy of STICA, to assess the durability of treatment response in these patients, and the impact on associated mental symptoms (for example social anxiety and depression).

### Outcomes

The primary efficacy endpoint is defined as improvement of IA/CA rated by the patient himself (primary outcome measure: AICA-S [30,31]). At the end of therapy an AICA-S score <7 indicates remission.

Secondary endpoints include the remission of IA/CA in the expert rating (AICA-C ≤ 13). The preoccupation with the internet or computer games will be analyzed (hours spent per week). IA and CA are associated with negative consequences in health, social communication, psychosocial wellbeing (GAF [28], BDI-II [30], LSAS [36]), level of performance in school or work, and self-efficacy (SWE [39]). For each instrument assessments at baseline will be compared to assessments obtained 4 and 6 months after therapy.

### Sample size calculation

The sample size calculation is based on the primary endpoint (T2: end of therapy) and a chi-square test without continuity correction on a two-sided level of significance of 0.05. The calculation is based on results from 33 patients, who participated in an open trial. Twenty-four patients improved according to the AICA-S < 7. A difference to the control group of 20% is considered as clinically relevant. With a power of 90%, 184 patients in total are needed to detect that difference. Considering the average therapy group size of eight, 16 subjects have to be randomized at the same time. Therefore, we will need to include 192 patients in this trial (*n* = 96 patients for each group). The primary analysis will be performed on the population of all randomized subjects (intention to treat (ITT) population). Subjects who discontinue the therapy will be regarded as non-improvers to treatment. Our previous experience with internet addicts revealed drop-out rates of about 27% (nine drop-outs from 33 patients).

### Randomization

Patients will be randomly assigned either to the STICA intervention group or to the WLC group. The randomization list will be generated stratified by the Interdisciplinary Centre for Clinical Trials (IZKS). Considering the average therapy group size of eight patients, 16 patients have to be randomized at the same time. The randomization ratio will be 1:1 within each center. After confirmation that a patient fulfils all inclusion criteria for randomization, the electronic case report form (eCRF) will immediately provide the investigator with the randomization results. Patients are subsequently informed about the randomization result and the intervention starts shortly after randomization. The IZKS will furthermore insure treatment integrity by regular site visits.

### Statistical analysis

#### Primary analysis

The primary efficacy endpoint is defined as the change of the AICA-S level. This will be analyzed using a logistic regression model with predictors of group (STICA treatment *vs.* WLC), pre-treatment score of AICA-S, education, trial center, and age.

The primary hypothesis to be tested is:

H_0_: π_STICA_ = π_WLC_*vs.* H_1_: π_STICA_ ≠ π_WLC_

where π_STICA_ and π_WLC_ are the probabilities to respond to treatment in the STICA treatment group and the WLC group, respectively. The primary analysis will be performed on the ITT population on a two-sided level of significance α = 0.05. The two-sided level of significance will be the same for all analyses. A completer analysis will be performed for sensitivity. Additionally, the analysis will be repeated with a predictor for the therapy group. Drop-outs during the treatment phase will be regarded as treatment failures.

#### Secondary analysis

The remission of IA/CA according to the AICA Checklist will be examined using logistic regression analyses with the same predictors as the primary analysis. The reduction of negative consequences, GAF, depression (BDI-II), and social anxiety (LSAS) will be examined using ANCOVA with covariates.

Analyses will be conducted on a two-sided level of significance of α = 0.05. Descriptive statistics are used to show changes over time. Serious adverse events and drop-outs will be analyzed by using descriptive statistics.

### Safety aspects

Safety parameters will comprise newly occurring psychiatric diagnoses (SCID-I [32]) and all serious adverse events that are reported during and up to 6 months after treatment. Therefore in the context of psychotherapy suicidal ideations or the global functioning level will be regarded.

### Medical complications

According to GCP, an adverse event (AE) is defined as follows: any untoward medical occurrence in a patient participating in a clinical trial. An AE can therefore be any unfavorable and unintended sign (including an abnormal laboratory finding), symptom, or disease, whether or not related to the trial intervention. Due to the fact that this trial analyses a psychological treatment, only AEs concerning psychological conditions, defined as any disorder classified by the International Classification of Diseases [48] F00-F99 (‘Mental and Behavioral Disorders’) will be documented.

For this study the following conditions were defined as AE: (1) new symptoms/medical conditions, (2) new diagnosis, (3) intercurrent diseases and accidents, (4) worsening of medical conditions/diseases existing before clinical trial start, (5) recurrence of disease, or (6) increase of frequency or intensity of episodical diseases.

A serious adverse event (SAE) is an AE that: (1) results in death, (2) is life-threatening, (3) requires patient hospitalization or prolongation of existing hospitalization, (4) results in persistent or significant disability/incapacity, or (5) is a congenital anomaly/birth defect.

All medical complications during the study are documented in the eCRF.

### Ethical issues

Clinical protocol and written informed consent were approved by the Ethics Committee (EC) of the Federal State of Rhineland Palatinate (Germany), which is responsible for the coordinating centre Mainz (Ref. No. 837.316.11 (7858)). Ethics Committees of all cooperating centers will provide the necessary additional documents.

All procedures described in the clinical trial protocol follow the ICH-GCP guidelines and the ethical principles described in the current revision of the Declaration of Helsinki. The trial will be carried out in keeping with local legal and regulatory requirements.

Before being admitted to the clinical trial, patients receive detailed explanations of the nature, scope, and possible consequences of the clinical trial in a form understandable to them. The patients must give consent in writing. Each patient will receive a copy of the signed informed consent document.

In this clinical trial all patients, including the WLC group will receive the full treatment. For the WLC patients the therapy begins after a waiting period of 4 months.

An independent Data Monitoring and Safety Board (DMSB) has been established for this study. The DMSB will supervise the conduct of this trial and will issue recommendations for early termination, modifications or continuation of the trial, if necessary. The DMSB and the EC must be informed immediately of study-related SAE.

## Discussion

The number of patients suffering from IA/CA who need professional help increases constantly. Up to now there is no specific manualized intervention program and there are no well-defined treatments of established efficacy. To our knowledge, STICA is the very first clinical trial for establishing efficacy of a specific treatment for IA/CA.

The efficacy of the treatment will be checked in a randomized controlled multicenter trial. The use of a WLC group appears to be justified because of the novel treatment approach and the lack of comparable approaches. Patients in the WLC are assured to receive full treatment after a waiting period of 4 months following randomization. Thus, however, follow-up of waitlist controls is not possible.

STICA will also regard co-morbid mental disorders and serious long-term consequences (for example social withdrawal or failure in school/education) caused by excessive internet or computer game use. The aim of STICA is the reintegration of the patients into a normal life, including controlled use of computer and internet, social contacts, and work performance.

The results of this study will be of high relevance because of the methodological demand and the high relevance of the topic. This study will determine the effectiveness and durability of a cognitive behavioral short-term treatment for IA/CA. For patient care it will be important to implement an effective treatment for IA/CA in clinical routine.

### Trial status

The first patient was enrolled to the STICA study on February 1, 2012. Follow-up measures for the last included patients were expected to be terminated in June 2014.

## Abbreviations

ACE = Adverse childhood experience questionnaire; AE = Adverse event; ADHD = Attention deficit hyperactivity disorder; AICA-S = Assessment of internet and computer game addiction, self report; AICA-Checklist = Assessment of internet and computer game addiction, expert rating; BDI-II = Beck Depression Inventory; CA = Computer game addiction; CDS-2 = Cambridge depersonalization scale; DFG = Deutsche Forschungsgemeinschaft; DMSB = Data Monitoring and Safety Board; d2 = Test of attention; EC = Ethics Committee; eCRF = Electronic Case Report Form; FLZ = Questionnaire of life satisfaction; GAF = Global Assessment of Functioning; GCP = Good Clinical Practice; HAQ = Helping alliance questionnaire; ICH = International Conference on Harmonisation of Technical Requirements for Registration of Pharmaceuticals for Human Use; IA = Internet addiction; ITT = Intention to treat; IZKS = Interdisciplinary Centre for Clinical Trials; LSAS = Liebowitz social anxiety scale; NEO-FFI = NEO Five factor inventory; PANAS = Positive and negative affective schedule; PHQ = Patient health questionnaire; PSS = Perceived stress scale; SAE = Serious adverse event; SCID = I/II Structured clinical interview for DSM IV; SCL-90-R = Symptom Checklist 90 revised; STICA = Short-term Treatment of Internet and Computer game Addiction; SWE = Assessment of the expectance of self-efficacy; WLC = Wait list control; WURS-k = Wender Uta rating scale.

## Competing interests

The authors declare that they have no competing interests.

## Authors’ contributions

SJ did the first draft of the manuscript and is contact person for questions about realization, design, and administration. SJ, MEB, and KW did the final draft of the manuscript and critically revised it for its intellectual content. KW and MEB developed the therapy, which will be evaluated with this study. The proposal was first prepared by KW, KWM, MEB, and CR. For the grant MEB and KW operate as principle and co-principle investigator. MEB is responsible for the proposal. KWM, CR, TW, KW, and MEB substantially contributed to the conception and the final design of the study. AB, MM, and KM are responsible for the correct realization of STICA in the different centers and cooperate to improve the study design. All authors read and approved the final manuscript.
